# Unusual Presentation of Epstein-Barr Virus-Associated Cholestatic Hepatitis in an Infant

**DOI:** 10.1097/PG9.0000000000000089

**Published:** 2021-06-15

**Authors:** Raafat Hammad Seroor Jadah, Noor Mohamed Ghassan Shaikho, Sara Abdulla Hasan

**Affiliations:** From the Department of Paediatrics, Bahrain Defence Force Royal Medical Services, Riffa, Bahrain.

**Keywords:** Epstein-Barr virus, transaminases, liver, cholestasis

## Abstract

Epstein-Barr virus (EBV) infections are prevalent in the pediatric population but are subclinical in the majority of cases. Elevated transaminases in the acute setting rarely increase beyond 5 times the normal upper limit. We present a girl aged 1 y with fever, vomiting, and diarrhea. Although initial physical examination was unremarkable, she developed jaundice, hepatomegaly, abdominal distension, and a maculopapular rash during admission. Laboratory investigations revealed marked increase in transaminases (alanine aminotransferase 7,664.5 IU/L, aspartate aminotransferase 12,266 IU/L), elevated γ-glutamyl transferase (224 IU/L), and hyperbilirubinemia (total 130.7 µmol/L, direct 104.9 µmol/L). Abdominal ultrasound reported hepatomegaly with mild ascites. Serology revealed that both Monospot test and EBV immunoglobulin G were positive. With supportive therapy, improvement was noted within a week of symptom onset. We hereby elucidate the importance of considering EBV as a cause of acute cholestatic hepatitis in a very young pediatric patient who develops a rapid elevation of liver enzymes.

## INTRODUCTION

Epstein-Barr virus (EBV) is a DNA virus that is common during childhood up to 18 y of age. Although children are usually asymptomatic, adolescents can present with symptoms of infectious mononucleosis. The clinical presentation of EBV is more prevalent with increasing age. In the acute phase, 90% of cases have a transient increase in hepatic transaminases with a favorable outcome, especially with appropriate symptomatic care and management (1). Rarely, however, patients may develop cholestatic hepatitis with jaundice being reported in 5% of cases (2,3).

## CASE REPORT

A previously healthy girl aged 1 y presented to the pediatric emergency department with a 5 d history of fever, vomiting, and diarrhea associated with reduced feeding and activity. There was positive history of eating outside.

Upon examination, the patient was febrile (38.9°C rectally), lethargic, drowsy, moderately dehydrated, and her Glasgow Coma Scale was 13 out of 15. Central nervous system, cardiovascular, and respiratory examinations were unremarkable. Her abdomen was soft, lax, nontender, with no distension, or organomegaly.

On the third day of admission, the patient became more lethargic and developed jaundice with a maculopapular rash on the trunk. Further examination showed abdominal distention with hepatomegaly but no splenomegaly. The liver edge was 3 cm below the costal margin with a total liver span of 10 cm. In view of the patient’s deterioration with a Glasgow Coma Scale 10 out of 15, she was shifted to the intensive care unit for further care.

## INVESTIGATIONS

Initial complete blood count, coagulation profile, liver function test, renal function test, and electrolytes were within normal limits.

At 24 h of admission, albumin dropped to 23.7 g/L (35–52 g/L) and liver enzymes started to rise: alanine aminotransferase (ALT) 52.9 IU/L (0–33 IU/L), γ-glutamyl transferase (GGT) 54 IU/L (5–36 IU/L), aspartate aminotransferase (AST) 72.7 IU/L (0–32 IU/L), total bilirubin 15.6 µmol/L (0–21 µmol/L), and direct bilirubin 12.4 µmol/L (0–5 µmol/L).

At 72 h of admission, albumin dropped further to 22.5 g/L and liver enzymes reached their peak: ALT 7,664.5 IU/L, GGT 224 IU/L, AST 12,266 IU/L, total bilirubin 130.7 µmol/L, and direct bilirubin 104.9 µmol/L. Additionally, the coagulation profile revealed international normalized ratio > 17 (0.98–1.13), partial thromboplastin time > 120 s (13–15 s), activated partial thromboplastin time 60 s (28–45 s).

Serological tests were positive for Monospot test, Epstein-Barr nuclear antigen immunoglobulin G (IgG) (60.1 U/mL [5–20]), and viral-capsid antigen IgG (VCA IgG) (144 U/mL [>20]). However, early antigen IgG and EBV immunoglobulin M (IgM) were both negative, 5 U/mL (10–40) and 10 U/mL (20–40), respectively. Other viral causes of hepatitis were ruled out due to negative antinuclear antibody, antidouble stranded DNA (anti-dsDNA), and antismooth muscle antibodies. Immunoglobulin G (IgG) was low (6.8 g/L [7–16]), while immunoglobin A (IgA) and IgM were within normal range, 0.79 (0.7–4) and 1.16 g/L (0.4–2.3), respectively. Moreover, paracetamol drug level was below threshold.

**FIGURE 1.**
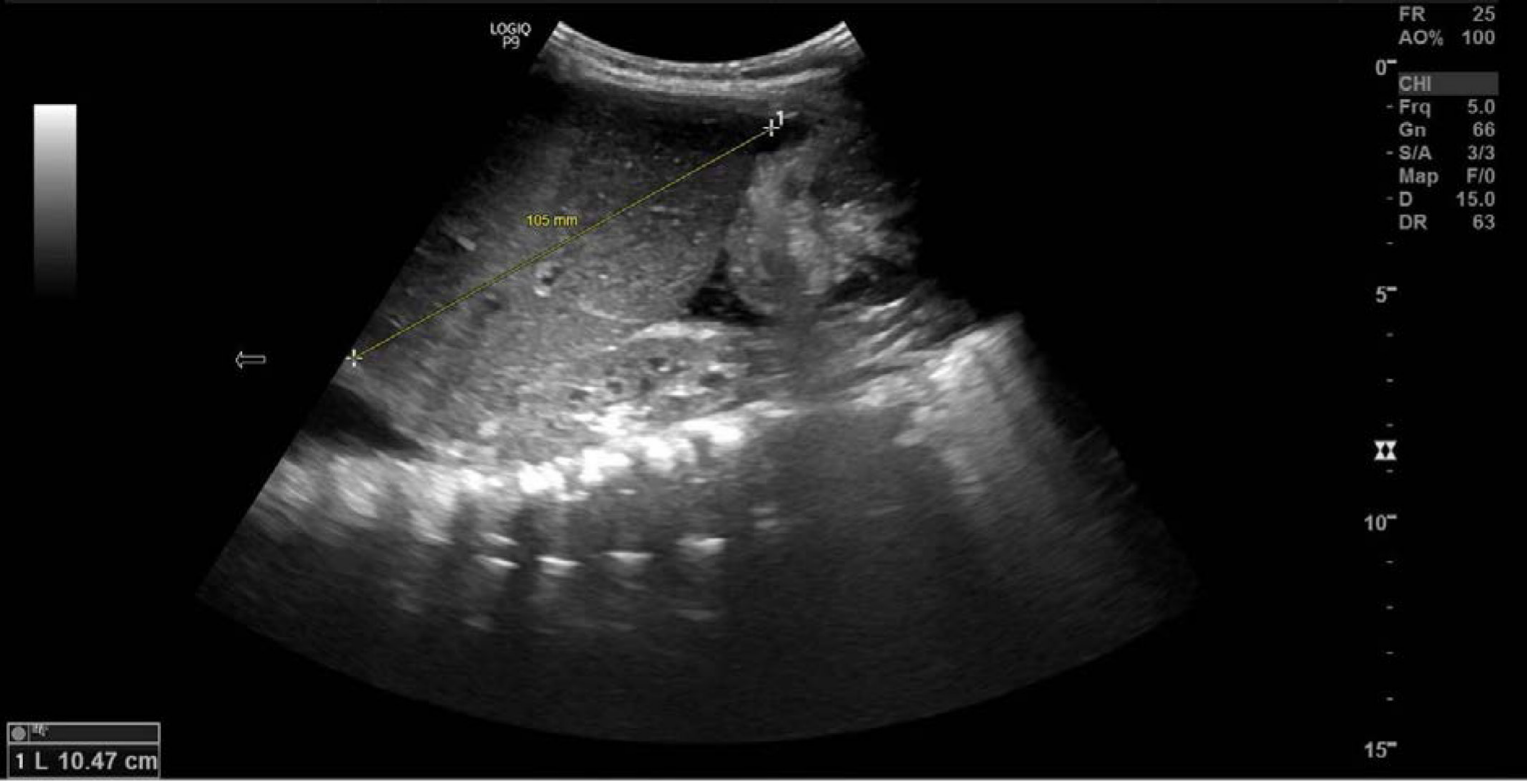
Abdominal ultrasound showing a 10.5 cm liver span along the midclavicular line. The liver has a homogenous texture and a smooth regular outline.

Additional investigations revealed negative blood culture and unremarkable stool routine microbiology and cytology. Abdominal ultrasound showed mild ascites, hepatomegaly (10.5 cm along the midclavicular line, Fig. 1), and biliary sludge.

## DIFFERENTIAL DIAGNOSIS

Initially, the clinical presentation was consistent with acute gastroenteritis. However, after the development of rapidly increasing transaminases with an abnormal coagulation profile, other differentials were considered.

The patient received paracetamol which may have caused paracetamol-induced hepatitis, but it was ruled out as its level was below threshold and the dose and frequency were appropriate (15 mg/kg every 6 h). Other viral causes for acute hepatitis (hepatitis A, B, C; cytomegalovirus; and herpes simplex virus) were negative on serological studies. Autoimmune hepatitis was suspected but later eliminated due to the negative antinuclear antibody, anti-dsDNA, antismooth muscle antibodies, as well as the presence of low IgG, normal IgM, and normal IgA.

Rare causes like CD 40 ligand deficiency was excluded as the patient did not have a history of recurrent infections or hospital admissions with the presence of normal IgM and IgA, low IgG, as well as a normal neutrophil count. Finally, although lymphoma was considered, the patient had no palpable lymph nodes, history of weight loss, or night sweats with a normal serial full blood count throught admission.

## TREATMENT

As the patient showed signs of moderate dehydration, 2 boluses of intravenous normal saline 0.9% (20 mL/kg) were given followed by maintenance intravenous fluids with an additional deficit of 10%. She was commenced empirically on Meropenem 200 mg (60 mg/kg/d) given intravenously 3 times a day for a total of 5 d due to the suspicion of an underlying infection.

The patient also received 1 unit of packed red blood cells as her hemoglobin dropped to from 97 to 76 g/L (120–160 g/L). She was also given 4 units of 100 mL fresh frozen plasma and vitamin K 5 mg once daily for a total of 5 doses to correct the coagulopathy. Subsequent coagulation profile revealed normal values.

Due to the development of bilateral lower limb edema, abdominal distention, and periorbital edema, she was given 1 dose of 20% Albumin solution 25 mL intravenously.

Commencing empirical N-acetyl cysteine for the management of potential paracetamol toxicity was not indicated as the patient was receiving the appropriate therapeutic dose with a below threshold paracetamol level.

## OUTCOME AND FOLLOW-UP

During the admission, the patient showed gradual clinical improvement following supportive management. Upon follow-up at the end of the second week, she showed complete clinical resolution with a normal abdominal examination and no hepatomegaly.

Repeated investigations on follow-up showed further regression in values with albumin 49 g/L, ALT 64.30 IU/L, GGT 89 IU/L, AST 72.80 IU/L, total bilirubin 28.30 µmol/L, and direct bilirubin 23.40 µmol/L.

## DISCUSSION

EBV is a DNA herpesvirus with an age-specific prevalence pattern. Prevalence increases with age; 50% in 6–8 y compared with 89% in 18–19 y (4). Only 5% of children between 3 mo and 18 y with elevated transaminases had an EBV infection (5).

Diagnosis of EBV is made based on serology for specific viral antibodies (VCA and anti-Epstein-Barr nuclear antigen) and heterophile antibodies (Monospot Test). VCA IgG is present in the primary infection and persistently stays positive, while VCA IgM may not be positive in the acute setting as it might appear 1–2 wks later (6).

In cases with isolated viral capsid antigen IgG, IgM may have not been detected due to either delayed or early seroconversion. This phenomenon is seen in 4.5% of children between 1 and 10 y. Consequently, children may have an isolated viral capsid antigen IgG in an acute EBV infection (7).

Although EBV infections are associated with elevated transaminases in the acute phase, they do not normally increase >5 times the upper limit of the normal range (2). About 80%–90% of patients with EBV have self-limiting mild hepatic involvement managed supportively (8). In our case, however, the hepatic transaminases reached a level not previously reported in literature with ALT over 7,000 IU/L and AST over 12,000 IU/L.

In a study conducted by Kofteridis et al, transaminase levels increased in the first week, peaked in the second week, and normalized in the third week after the onset of the infection (8). Our patient experienced a rapid rise in transaminases reaching its peak within 72 h from admission followed by a gradual decline and complete normalization by the end of the second week.

In conclusion, EBV-associated cholestatic hepatitis should be considered in a very young patient with significantly elevated hepatic transaminases. Our patient presented at the age of 1 with marked elevation in liver enzymes, not previously reported in the literature. Early diagnosis and management with supportive care are associated with a favorable outcome.
